# Trends in Heart Disease Burden and Risk Factors in California's High-Risk Populations

**DOI:** 10.1016/j.jaccas.2025.105653

**Published:** 2025-10-07

**Authors:** Ryan Nazari

**Affiliations:** Independent Researcher, Modesto, California, USA

**Keywords:** California, cardiovascular disease, health disparities, prevention programs, socioecological model

## Abstract

**Background:**

Heart disease is the leading cause of death in the United States. California's overall rates are lower than the national average, but gaps persist for high-risk communities.

**Project Rationale:**

Mortality rates increased during the COVID-19 pandemic, reversing decades of progress, and many existing programs do not address deeper determinants of health.

**Project Summary:**

Using the socioecological model and a multimethod secondary analysis, this review examined California heart disease trends, risk factors, and programs from 2018 to 2022. Mortality rose in California and the U.S., with men and older adults having the highest rates. Programs improved individual awareness but often lacked community and policy integration.

**Take-Home Message:**

Equity-focused, community-based prevention backed by stable funding and policies is essential to reduce health disparities in cardiovascular disease.

Heart disease causes about 695,000 deaths each year in the United States.^1^ California often performs better than the national average, but statewide numbers can mask significant differences across counties and groups.^2^ These disparities connect to access to primary care, the places people live and work, and social conditions that shape choices and stress.^3^^,^^4^ From 2018 to 2022, heart disease mortality rates in California rose after years of decline.^2^ This report uses the socioecological model to review trends, key risk factors, and how well current programs reach high-risk communities in California.

**Take-Home Messages**

• California needs community-based heart disease prevention efforts that are easy to reach and culturally informed.
• Durable progress depends on policies and funding that support prevention and follow-up care.

## Case Summary Prompting the Project Launch

During the COVID-19 pandemic, county reports showed steeper rises in mortality in places that had already struggled with access. In several rural areas, people reported long drives for routine blood pressure checks and limited clinic hours. In dense urban neighborhoods, residents described crowded housing, food insecurity, and inconsistent internet service that made remote visits hard. Local teams held pop-up events in parking lots and community centers, but many people could not return for follow-up. These observations raised a guiding question: Are current prevention and treatment efforts reaching people who most need them, and if not, which gaps should be fixed first?

## Project Rationale

California looks strong on paper, but progress is not shared equally. High-risk populations face stacked barriers that include cost, transportation, language, and trust in the system.^3^ Local conditions such as food access and safe spaces to be active also play a role.^4^ Many programs aim at personal behavior change, yet they do not always address the surroundings that drive risk or the policies that could lower it over time.^5^ A structured review can help health systems and community partners direct resources toward prevention that lasts and meets people where they are.

## Project Description

### Framework

The socioecological model was used to organize findings at the individual, interpersonal, community, and policy levels.

### Design

A multimethod secondary analysis blended a targeted review of peer-reviewed and gray literature with descriptive analysis of public data.

### Data sources

The Centers for Disease Control and Prevention's WONDER (Wide-ranging ONline Data for Epidemiologic Research) database was the source for mortality data, the California Health Interview Survey was used for behavior and access, and the Behavioral Risk Factor Surveillance System was used for risk factor prevalence; additional data sources included reports and tools from the Centers for Disease Control and Prevention and the California Department of Public Health.

### Inclusion

Included were sources from the past 10 years that focused on California or comparable United States settings and addressed high-risk populations.

### Variables

The main outcome was age-adjusted mortality from heart disease per 100,000 residents. Key risk factors included hypertension, obesity, tobacco exposure, and diabetes. The county context used median household income as a simple proxy for resources.

### Quantitative Approach

Mortality and risk factor trends were summarized by year, sex, and age group. For county comparisons, the most recent 3 years were averaged to smooth random swings. Very small counties were treated as descriptive rather than for ranking. Trends in California and the United States were graphed side by side to illustrate the different levels and similar shapes of the recent rise.^2^

### Qualitative Approach

Programs targeting high-risk communities were coded according to socioecological model level. For each, the review recorded who the program tried to reach, how enrollment worked, whether materials were in preferred languages, and whether there was a clear path to a clinic visit for follow-up. Themes were grouped across levels. A theme had to appear in at least 2 sources to be treated as a finding, and county patterns were compared with the literature. The goal was to provide an actionable map rather than to test a single hypothesis.

## Project Deliverables

The project produced 4 outputs: 1) a county table pairing mortality with median income to show where gaps are largest and where exceptions exist; 2) a summary of risk factors and social conditions at each socioecological model level to guide clinic and community planning; 3) examples of promising practices that could translate to California, such as integrated hubs offering primary care, nutrition support, mental health access, and help with benefits in a single location; and 4) recommendations aligned with financing and workforce constraints so pilots can launch within a year.

## Project Outcome, Impact, and Future Directions

Mortality differences often track with income, but access to care, local infrastructure, and culture also influences results. In the county table, higher income areas such as Santa Cruz showed lower mortality, whereas lower income areas such as Fresno and Imperial showed higher mortality, with exceptions that prompted a closer look. For example, Marin had a high income but a mortality rate of approximately 89 per 100,000; Santa Cruz was close to 55 per 100,000; and Fresno was above 100 per 100,000. California as a whole rose from about 141 per 100,000 in 2018 to a peak of approximately 148 in 2020 and 2021, then dipped slightly in 2022.^2^ Men and adults older than 65 had the highest mortality.^6^ In high-risk groups, hypertension and related conditions were common.^7^ Programs with promise included mobile clinics that brought screening and coaching to farms and fairgrounds, worksite wellness programs with on-site blood pressure checks, and strong tobacco control paired with plain language education.^5^^,^^8^

These efforts raised awareness, and patients started treatment earlier. Limits appeared when events were one-time only, when materials were not in preferred languages, or when there was no easy path to a clinic for follow-up. A durable path means a visit is scheduled before a person leaves an event, transportation is arranged when needed, and a community health worker calls to confirm.

A practical plan could launch in 12 months:

• Months 0 to 3: Form a steering group from the health department, a community clinic, a school district, and a trusted community group; pick 2 priority zip codes using recent mortality and blood pressure control data.
• Months 3 to 6: Set a rotating schedule for a mobile team with 2 stops per week in each ZIP code and train community health workers to do blood pressure checks, tobacco counseling, and appointment scheduling on site.
• Months 6 to 9: Start a produce prescription pilot program with local stores and extend evening clinic hours once per week.
• Months 9 to 12: Track percentage with controlled blood pressure, follow-up completion within 30 days after an event, and no-show rates for new visits, then adjust routes, hours, and messages based on the data and resident feedback.

## Discussion

The findings align with what is known about social and structural drivers of cardiovascular risk.^1^^,^^3^ Education and coaching help, but larger and more durable gains come when prevention is linked to stable community resources and policy support that removes practical barriers such as cost and transportation.^5^ Examples from other regions suggest that hubs that combine primary care with nutrition support and mental health access can reduce gaps in control of risk factors. California can build on community clinics and county programs to create similar hubs, especially if payment models cover community health workers and include prescription coverage.

## Limitations

This study used secondary data and a targeted review rather than field work. Small counties can have unstable rates from year to year, so 3-year windows were used, and the smallest values were treated as descriptive. Because some race and ethnicity details were not consistently available, the review focused on geography, age, sex, and social conditions that affect many groups. Even with these limits, the patterns point to practical next steps that can be tested quickly.

## Conclusions

California can keep overall mortality low and still miss people at the highest risk of heart disease. Closing that gap requires prevention that meets people where they live, respects language and culture, and is backed by policies and funding that last. This combination is the most realistic way to reverse recent increases and make progress that holds over time.

## Funding Support and Author Disclosures

The author has reported that there are no relationships relevant to the contents of this paper to disclose.
